# St. Louis Medical and Surgical Journal

**Published:** 1847-08

**Authors:** 


ARTICLE VII.

### ST. LOUIS MEDICAL AND SURGICAL JOURNAL.

Our Exchange bearing the above title, having completed its fourth volume, comes to us in a new and more beautiful dress and is hereafter to be issued every other month. The number before us (No. 1, Vol. V.) contains one hundred and four pages of interesting matter.

We hope it will find a justification of the additional pains with which it is got up, in a liberal extension of patronage.

